# *Pseudomonas cremoricolorata* Strain ND07 Produces *N*-acyl Homoserine Lactones as Quorum Sensing Molecules

**DOI:** 10.3390/s140711595

**Published:** 2014-06-30

**Authors:** Nina Yusrina Muhamad Yunos, Wen-Si Tan, Chong-Lek Koh, Choon-Kook Sam, Nur Izzati Mohamad, Pui-Wan Tan, Tan-Guan-Sheng Adrian, Wai-Fong Yin, Kok-Gan Chan

**Affiliations:** 1 Division of Genetics and Molecular Biology, Institute of Biological Sciences, Faculty of Science, University of Malaya, 50603 Kuala Lumpur, Malaysia; E-Mails: ninayusrina@hotmail.com (N.Y.M.Y.); tmarilyn36@gmail.com (W.-S.T.); zetty_mohamad@yahoo.com (N.I.M.); acelinetan38@yahoo.com (P.-W.T.); adrian_tan_1991@yahoo.com (T.-G.-S.A.); yinwaifong@yahoo.com (W.-F.Y.); 2 Natural Sciences and Science Education AG, National Institute of Education, Nanyang Technological University, 1 Nanyang Walk, Singapore 637616, Singapore; E-Mails: chonglek.koh@nie.edu.sg (C.-L.K.); choonkook.sam@nie.edu.sg (C.-K.S.)

**Keywords:** cell-to-cell communication, mass spectrometry, triple quadrupole liquid chromatography mass spectrometry, *N*-octanoyl-l-homoserine lactone (C8-HSL), *N*-decanoyl-l-homoserine lactone (C10-HSL), *Pseudomonas cremoricolorata*

## Abstract

Quorum sensing (QS) is a bacterial cell-to-cell communication system controlling QS-mediated genes which is synchronized with the population density. The regulation of specific gene activity is dependent on the signaling molecules produced, namely *N*-acyl homoserine lactones (AHLs). We report here the identification and characterization of AHLs produced by bacterial strain ND07 isolated from a Malaysian fresh water sample. Molecular identification showed that strain ND07 is clustered closely to *Pseudomonas cremoricolorata*. Spent culture supernatant extract of *P. cremoricolorata* strain ND07 activated the AHL biosensor *Chromobacterium violaceum* CV026. Using high resolution triple quadrupole liquid chromatography-mass spectrometry, it was confirmed that *P. cremoricolorata* strain ND07 produced *N*-octanoyl-l-homoserine lactone (C8-HSL) and *N*-decanoyl-l-homoserine lactone (C10-HSL). To the best of our knowledge, this is the first documentation on the production of C10-HSL in *P. cremoricolorata* strain ND07.

## 1. Introduction

Quorum sensing (QS) is a term coiled by Fuqua and co-workers referring to a bacterial cell-to-cell communication system used as a tool to control their physiological processes [1]. The regulation of specific activity is dependent on the concentration of the cell population which is synchronized by the signaling molecules produced. Virulence factors are known to be the best-documented activity regulated by QS [2,3]. The signaling molecules responsible for the QS action is known as autoinducers which are classified into two major groups, the autoinducer-1 group such as the *N*-acyl homoserine lactones (AHLs) which are commonly utilized by Gram-negative bacteria, and oligopeptides classified as autoinducer-2, utilized by Gram-positive bacteria [4,5].

To date, the most commonly studied autoinducers are the AHL molecules produced by most Gram-negative bacteria. The AHL molecule side chain length varies from short chain (C4) to long chain (C18) carbon chains [6]. When at a higher population size, AHLs accumulate and bind to the AHL receptor protein forming the AHL-receptor complex which is then an active regulator that regulates QS-dependent genes. This QS system involves the LuxI-LuxR complexes where LuxI is AHL synthase and LuxR is the AHL receptor protein [7,8]. Once the production of AHL has exceeded the threshold level, the AHL/LuxR complex will then be in an active form and be able to regulate QS genes. This will then allow the QS bacteria to synchronize the gene regulation in a cell density-dependent manner allowing group behaviors in unison. This will give QS bacteria a competitive advantage allowing them to overcome many challenges, which is impossible for a few cells but easy with a population of cells.

QS bacteria have been isolated mainly from clinical and some other habitats [4,5] while there is still not much work done on the QS properties of fresh water-borne bacteria. Other than that, the study of QS could be beneficial in biotechnology whereby QS-based engineered genetic devices could perform a wide array of functions such as controlling gene expression, population and maintenance of synthetic ecosystems [9]. The knowledge about QS could have an impact in regulating bacterial phenotypes thus inhibition of QS represents a strategy which has also been applied to, for example, engineered plants which results in plants more resistant to infection by common pathogens such as *Pseudomonas syringae* and *Erwinia carotovora* [9,10]. Fresh water could be a potential environmental reservoir for various bacteria [11] and hence it raised our interest in isolating bacteria that possess QS properties from fresh water. It is the aim of this study to acquire isolates, and study the AHL production by *Pseudomonas cremoricolorata* strain ND07.

## 2. Experimental Section

### 2.1. Collection of Water Samples

The collection site of water samples was at the Sungai Ampang waterfalls, Malaysia (GPS coordinate: N 03°12.69′ E 101°47.72′) in 2013. Water samples were collected at the depth of 12–17 cm using sterilized plastic tubes. The temperatures and pH of the samples were recorded. Water samples were kept in an icebox and brought to the laboratory for further analysis [12].

### 2.2. Isolation of Bacterial Strains

Overnight culture was serially diluted and plated on Reasoner's 2A (R2A) agar [13]. The plates were incubated at 28 °C for 24 h. After 24 h, any visibly distinct bacterial colonies were identified and each colony was transferred by streaking onto Trypticase Soy Agar (TSA) followed by incubation (28 °C for 24 h). The color, shape and size as well as other visual properties of the colonies were recorded.

### 2.3. Strain Identification Using 16S rDNA

The QIAamp^®^ DNA Mini Kit (Qiagen, Germantown, MD, USA) was used to extract and purify bacterial genomic DNA of strain ND07 and the resulting DNA was used as template for PCR. The forward primer 27F and the reverse primer 1525R were used to amplify the 16S rDNA gene [14]. The PCR condition used in this study was as previously reported [15]. GenBank databases using the BLASTN program were used to compare gene sequences followed by sequence alignment and phylogenetic analyses using the Molecular Evolutionary Genetic Analysis (MEGA) version 6 [14].

### 2.4. Detection of AHL

An AHL biosensor, namely *Chromobacterium violaceum* CV026, was used to detect the presence of short chain AHLs [16] whereby CV026 cells will respond by forming a purple pigmentation [14,16] on Luria Bertani (LB) agar. *Erwinia carotovora* GS101 were used as positive control while *Erwinia carotovora* PNP22 was negative control [17,18].

### 2.5. Extraction of AHLs

The bacterial colony of strain ND07 showing positive results for the detection of AHL was grown overnight in LB broth buffered to pH 6.5 with 3-(*N*-morpholino)propanesulfonic acid (MOPS, 50 mM, pH 6.5) at 28 °C with shaking (220 rpm). An equal volume of acidified (0.1% v/v acetate acid) ethyl acetate was used to extract twice the spent supernatant [19]. The AHL extracts were then dried in a fumehood and stored at −20 °C for further analysis using liquid chromatography-mass spectrometry.

### 2.6. AHL Identification by Triple Quadrupole Liquid Chromatography Mass Spectrometry (LC/MS)

Extracted AHLs were resuspended in acetonitrile followed by LC/MS analysis using an Agilent 1290 Infinity LC system (Agilent Technologies, Santa Clara, CA, USA) equipped with an Agilent ZORBAX Rapid Resolution High Definition SB-C18 Threaded Column [20]. Mass spectrometry parameters and mobile phases used were essentially performed according to a reported method [20]. Precursor ion-scanning analysis were performed in positive ion mode with Q3 set to monitor for *m*/*z* 102 and Q1 set to scan a mass range of *m*/*z* 80 to 400. A molecular mass of *m*/*z* 102 is characteristic of the lactone ring moiety thus indicating presence of AHLs. The Agilent Mass Hunter software was used to analyse the MS data in order to confirm the presence of AHLs [20]. Analysis was based on the retention index and the comparison of the EI mass spectra with AHL standards was carried out.

### 2.7. Biofilm Assay

The biofilm assay was performed as described previously [21,22]. Briefly, the overnight culture of strain ND07 was adjusted to OD_600_ of 0.1, and the diluted culture was added with LB medium supplemented with catechin (1 mg/mL, anti-QS compound) [1] in a microtitre well. ND07 cultures treated with and without DMSO served as negative and positive controls, respectively. The ND07 cells with different culture conditions were incubated statically for 36 h at 28 °C. The planktonic bacteria were removed by washing thrice with sterile distilled water [3] and the plate was air-dried for 15 min followed by staining with 200 μL of 0.1% (w/v) crystal violet for 30 min. After staining, the excess crystal violet was removed and washed with sterile distilled water twice. The quantitative analysis of biofilm production was done by adding 200 μL of 95% (v/v) ethanol and 100 μL of the resulting solution was transferred to a new microtitre plate. The absorbance of the solution was read at OD_590_ with microplate reader. All experiments were performed in triplicate.

## 3. Results and Discussion

### 3.1. Identification of Bacteria Isolates

**Figure 1.**
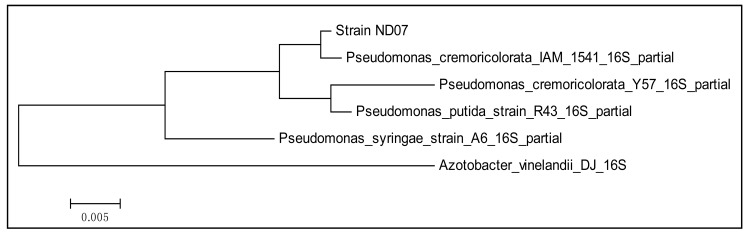
The evolutionary history was inferred by using the Maximum Likelihood method based on the Tamura-Nei model. The tree is drawn to scale, with branch lengths measured in the number of substitutions per site. All positions containing gaps and missing data were eliminated. Evolutionary analyses were conducted in MEGA6.

A pure colony of strain ND07 was obtained after several successive streakings. Physically the strain ND07 colonies were smooth, entire, flat to convex and creamy yellow in color. Strain ND07 was identified using 16S rDNA gene nucleotide analysis, which showed 99% similarity to *Pseudomonas cremoricolorata* strain IAM 1541. The result obtained was based on a web search and phylogenetic analysis of 16S rDNA nucleotide sequence (Figure 1). A total of 975 positions in the final dataset was used for analysis (Figure 1). All the characteristics of *Pseudomonas cremoricolorata* are in agreement with the reported work by Uchino and colleagues [23].

### 3.2. Detection of AHLs

*Pseudomonads* are ubiquitous Gram-negative bacteria which live in several environmental niches and have the ability to undergo transitions to become dangerous human pathogens [24]. Many *Pseudomonads* play roles as plant pathogens and also as opportunistic human pathogens [25]. According to Whiteley and colleagues, *P. aeruginosa* produces two different AHLs known as 3-oxo-C12-HSL and C4-HSL. Other members of the *Pseudomonads*, such as *P. syringae* produce 3-oxo-C6-HSL [26], *P. chlororaphis* produces C6-HSL [27] and *P. fluorescens* produces long chain AHLs [28].

**Figure 2.**
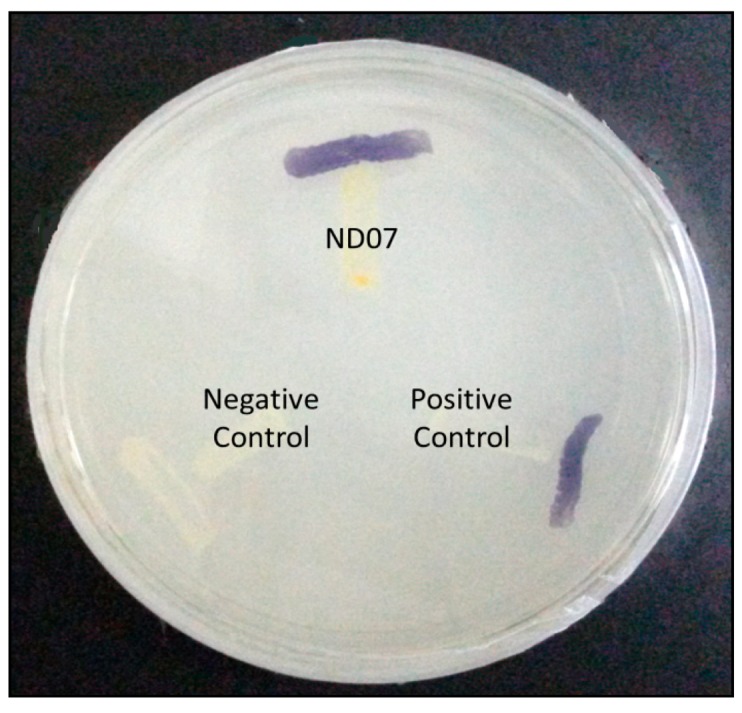
Detection of *P. cremoricolorata* (ND07) AHL production with *C. violaceum* CV026. Purple pigmentation shows the production of short chain AHLs. *E. carotovora* GS101 (Positive control) and *E. carotovora* PNP22 (Negative control) were included as controls.

**Figure 3.**
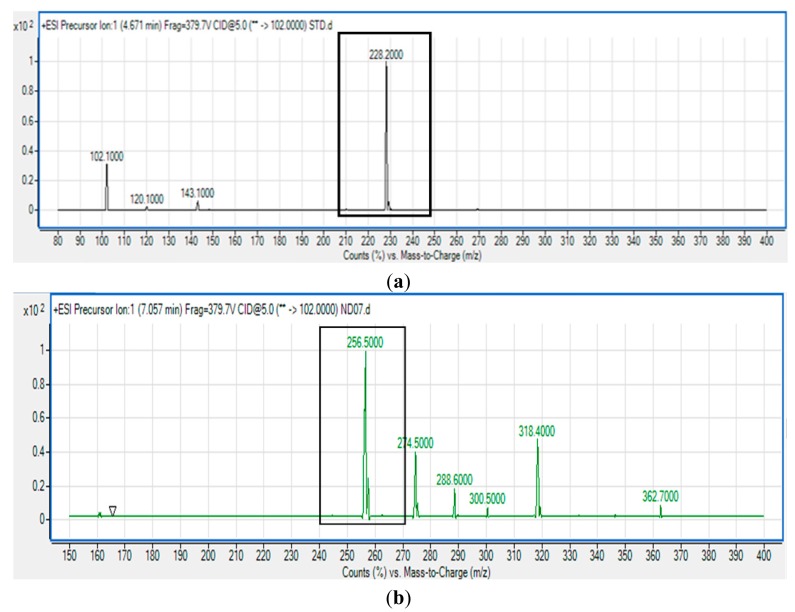
Mass spectra of AHL extract of the spent supernatant of *P. cremoricolorata* strain ND07 by triple quadrupole LC/MS which confirmed production of (**a**) *N*-octanoyl-l-homoserine lactone (*m*/*z* 228.2000); (**b**) *N*-decanoyl-l-homoserine lactone (*m*/*z* 256.5000) (boxed).

*P. cremoricolorata* strain ND07 was screened for the production of AHLs by using *C. violaceum* CV026. Strain ND07 showed positive results by triggering violacein production in *C. violaceum* CV026, suggesting the production of short chain AHLs (Figure 2). To further verify the AHL production, we used high resolution triple quadrupole LC/MS. The presence of AHLs were confirmed by the mass spectrometry analysis, and specifically *N*-octanoyl-l-homoserine lactone (C8-HSL) (*m*/*z* 228.2000) and *N*-decanoyl-l-homoserine lactone (C10-HSL) (*m*/*z* 256.5000) were detected (Figure 3).

The MS results shown in Figure 3 confirmed the presence of C8-HSL and C10-HSL in the extract of spent culture supernatant of *P. cremoricolorata* strain ND07, which is the first report of such a finding. According to Uchino and colleagues, *P. cremoricolorata* sp. nov. has been identified as a new species [23]. Our work on *P. cremoricolorata* will pave a way to a deeper understanding of the QS system in this bacterium.

### 3.3. Biofilm Formation of Enterobacter sp. Strain ND07

**Figure 4.**
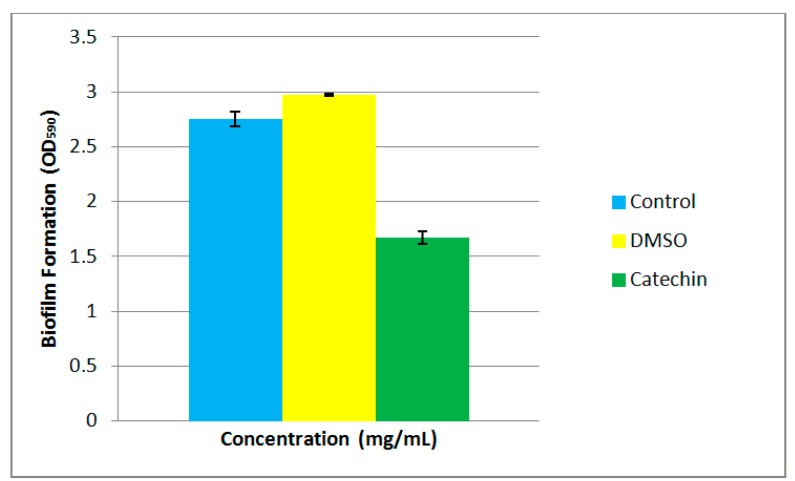
Qualitative analyses of violacein inhibition by anti-QS compound, catechin. Bars: standard errors of the mean. Solvent of catechin served as control.

Biofilm formation is often a QS-regulated phenotype [29,30]. The formation of biofilms often initiates with bacteria colonization, followed by surface attachment and finally biofilm development and maturation. The ability to form bacterial biofilms is often linked to pathogenic traits during chronic infections. There are several reports that members of *Pseudomonas* possess the ability to form biofilms [31]. *P. cremoricolorata* strain ND07 has been shown to be able to form biofilms (Figure 4). In our study, we demonstrated that the biofilm formation by strain ND07 can be interrupted by catechin, an anti-QS compound [21] and this suggests that biofilm formation by strain ND07 could be a QS-dependent phenotype in *P. cremoricolorata* strain ND07 (Figure 4).

Since QS regulates a battery of bacterial virulence factors [32], hence this work illustrated the significance in expanding the research on AHL-producing bacteria present in environmental samples. We are currently conducting whole genome sequencing on *P. cremoricolorata* strain ND07 aiming to study the AHL synthase and receptor genes that will provide more insight into the QS regulatory system in this bacterium.

## 4. Conclusions/Outlook

In conclusion, *P. cremoricolorata* strain ND07 showed QS activity with the production of C8-HSL and C10-HSL according to LC/MS analysis. To the best of our knowledge, this is the first report of such a finding. *P. cremoricolorata*, ND07 has also been shown to form biofilms which could be inhibited by anti-QS compounds, suggesting that it is QS-dependent trait. Our group is currently performing whole genome sequencing in order to identify the *luxI*/*luxR* genes in this isolate.
